# Sex-Related Differences in Allelic Frequency of the Human Beta T Cell Receptor SNP rs1800907: A Retrospective Analysis from Milan Metropolitan Area

**DOI:** 10.3390/vaccines9040333

**Published:** 2021-04-01

**Authors:** Elena M. Varoni, Giovanni Lodi, Massimo Del Fabbro, Andrea Sardella, Antonio Carrassi, Marcello Iriti, Pasquale Tripputi

**Affiliations:** 1 Dipartimento di Scienze Biomediche, Chirurgiche ed Odontoiatriche, Università degli Studi di Milano, Via Beldiletto 1, 20142 Milano, Italy; elena.varoni@unimi.it (E.M.V.); giovanni.lodi@unimi.it (G.L.); massimo.delfabbro@unimi.it (M.D.F.); andrea.sardella@unimi.it (A.S.); antonio.carrassi@unimi.it (A.C.); 2 National Interuniveristy Consortium of Materials Science and Technology (INSTM), 50121 Firenze, Italy; 3 IRCCS Orthopedic Institute Galeazzi, 20161 Milano, Italy; 4 Department of Agricultural and Environmental Sciences, Università degli Studi di Milano, 20142 Milano, Italy; 5 BAT Center—Interuniversity Center for Studies on Bioinspired Agro-Environmental Technology, University of Napoli “Federico II”, Portici, 80055 Napoli, Italy

**Keywords:** genotype distribution, polymorphism, heterozygosis, homozygosis

## Abstract

This paper aims at retrospectively re-analyzing the different distribution, between males and females, in the allelic frequency of the human β T cell receptor (TCR β) single nucleotide polymorphism (SNPs) rs1800907 in Caucasian patients in the Milan metropolitan area. The allelic frequency significantly differed between sexes. Females showed higher frequency of C/C genotype than males, but lower T/C genotype (*p* < 0.0001). Heterozygous (T/C) versus homozygous (T/T + C/C) genotypes resulted in a different distribution of frequencies in males than in females, the latter possessing higher homozygosis (*p* < 0.0001). Within the limitations of this work (small number of included studies that concerned just a specific geographical area), allelic distribution according to sex might account the role of TCRβ-related SNPs in autoimmune diseases and further investigations are required to explain better this genetic background, in the perspective of a sex-related T cell immune responsiveness and auto-immunity.

## 1. Introduction

Humans, similarly to several other vertebrate species, are characterized by sex-specific genes, which accounts for differences between males and females in a plethora of anatomical, pathophysiological, and behavioral traits [1]. This aspect can also be related to the response of individuals in leading to inflammation and immune activation. Females show higher immune response to infections, but they suffer more from autoimmune diseases. The pathophysiology of autoimmune and infectious diseases are, indeed, regulated by sex-linked factors, including sex hormones (testosterone shows an immunosuppressive effect, while estrogen has an immunoenhancing effect) and sex-chromosome-encoded genes (e.g., gene encoding for FoxP3, CD40L, TLR7, TLR8, and IL2 receptor subunit gamma are located on the X chromosome) [2,3]. During pregnancy, an enhanced network of immune response characterizes the maternal immune system, which is ready to trigger immunity, in case, to assure the fetal and maternal well-being [4]. Sexual dimorphism in immunity can lead to a more robust immune response in women, but to higher predisposition to autoimmune disorders. As an example, a growing number of findings support the idea that sex- and gender-related factors may be implicated in coronavirus disease 2019 (COVID-19) susceptibility, revealing higher fatality rates in men than in women [5]. On the other hand, many human autoimmune diseases, such as systemic lupus erythematosus multiple sclerosis and autoimmune thyroid disease, have an increased incidence and prevalence in females, while just a few others, including autoimmune diabetes, Guillain Barré syndrome (GBS) and psoriasis, are more prevalent in males [6]. Therefore, gender differences, which exist in infectious diseases and in autoimmunity, follow a male and a female bias, respectively [7].

T lymphocytes, in particular, control immune response by means of a highly variable T cell receptor (TCR) composed of two polypeptide subunits, α TCR subunit (TCRα) and the β subunit (TCRβ). The expression of TCRs on the surface of T lymphocytes is crucial for the establishment of T cell-mediated adaptive immunity. TCR antigen recognition is dependent on the presentation of short antigen peptides by the major histocompatibility complex (MHC) proteins expressed on the surface of other cells. The direct molecular interaction between the peptides, i.e., the MHC complex and the TCR, greatly influences TCR gene usage and has a fundamental part in shaping T cell/expressed TCR repertoires, particularly during T cell maturation and differentiation [8]. TCR genes are located at three regions in the genome, 14q11.2 (TCR α, δ), 7q34 (TCR β) and 7p14 (TCR γ). Each of these regions includes sets of related gene segments that are somatically recombined to form transcripts that ultimately code for α/β or β/γ TCR heterodimeric protein chains [8]. The potential impact that sex hormones could have on T-lymphocytes may explain, partly, female endurance against infections and predisposition to autoimmune diseases, independently from genetics, via both receptor-mediated and receptor-independent pathways. Lymphocytes cells have, in addition, androgen, progesterone and estrogen receptors that modify immune response to infections and susceptibility to autoimmune disease [9]. Estrogen, in particular, can promote Th2 response, inducing T cell homing by enhancing the expression of the homing marker CCR5, while androgens increase Th1 response and activation of CD8 cells, down-regulating natural killer (NK) cell response, tumor necrosis factor-alpha (TNFα), and increasing the production of anti-inflammatory IL-10 [5]. Considering autoimmunity, a sex-specific response was observed in humanized murine models of inflammation, revealing that the exogenous estradiol and castration in male mice led to an increase in autoimmunity by augmenting MHC-II expression and modulating B cells function [5]. This major role of sex hormones is the main cause of sex-biased autoimmune diseases, including rheumatoid arthritis.

From a genetic point of views, a plethora of polymorphisms, as single nucleotide polymorphisms (SNPs), in TCRβ has been analyzed, to date. Although some of them have been associated with changes in both expression and function of the TCRβ gene and with the progression of certain autoimmune diseases, such as in rheumatoid arthritis, the power associating variants in this locus and the disease susceptibility remain still controversial [10].

The full comprehension of genotype distribution between males and females represents a pivotal issue to understand the physiological and pathogenic role of TCRβ-related SNPs in health and disease. The SNP rs1800907, in the promoter region of the constant region of TRCβ, has been extensively studied in the nineties and it plays a pivotal role in the immune response. The SNP rs1800907 has been associated with several T cell-mediated autoimmune diseases, most of them more likely to affect women. These included insulin-dependent diabetes [11], autoimmune hepatitis [12], IgA nephropathy [13], membranous nephropathy [14], Graves’ disease and Hashimoto’s thyroiditis [15]. In all these studies, the Southern blot analysis of restriction fragment length polymorphisms (RFLP), obtained by cutting genomic DNA with restriction enzyme Bgl II, was performed. Since 2000, when more accurate new techniques, including polymerase chain reaction (PCR) followed by direct automatic sequencing, became available, the nucleotide sequence (T/C) and genotypic frequencies of this SNP (T/C, T/T, C/C) have been defined in healthy individuals and patients from the Milan metropolitan area [16,17,18]. A further study from the same group found that heterozygous patients resulted in less severe primary biliary cirrhosis (PBC), and the difference in the disease’s clinical stage became statistically significant when patients with T/C and C/C genotypes were compared [19]. A female-bias is also present in this case, since the prevalence of PBC, which in the last decade increased to 34.6 per 100,000 inhabitants, shows a female:male gender ratio of 4:1 [20]. On the other hand, some authors have argued that a considerable number of associations between disease genetic markers and disease phenotypes, including diabetes and obesity, were highly unreproducible [21,22].

This paper aims at re-analyzing, retrospectively via the aggregation of datasets coming from different studies conducted in the Milan metropolitan area, the diverse distribution of the TCRβ SNP rs1800907 allelic frequency between males and females, while being aware of the intrinsic limitations related to the retrospective design and to the limited source of data, coming from a single geographical provenance.

## 2. Materials and Methods

### 2.1. Studies Included for Data Aggregation

Six studies (case-control and single-cohort, prospective and retrospective studies) already published [16,17,18,19,23] were included for the retrospective re-analysis. Each study was conducted in humans, and investigated the allelic frequency related to the TCRβ SNP rs1800907 in Caucasian patients coming from the Milan metropolitan area.

### 2.2. Study Participants 

The population was composed of those subjects enrolled in the included studies, both cases and healthy controls. All individuals were Caucasian and recruited from the single metropolitan area of Milan (Italy) after informed consent, and were over the age of 18 years old.

### 2.3. Allelic Frequency

Allelic frequencies (T versus C alleles; T: 0-5427 versus C: 0-4573) were tested for sex-related differences, thus comparing female and males (independently from being healthy subjects or cases), as explained in detail in each of the included studies. Briefly, DNA is extracted from 5 mL of peripheral blood by proteinase K digestion, phenol extraction and ethanol precipitation as previously described [24] (Tripputi, Guérin and Moore, 1988). PCR is performed with 40 cycles of 93° for 1 min, 55° for 1 min and 72° for 1 min and carried out with primers designed around the polymorphic Bgl II site, according to sequences of the NCBI program (GenBank accession number U66061, OMIN number 186930).

Forward primer: 5′-TAATTTTGAAATAAGGGAAGATGAC-3′
Reverse primer: 5′-TTTTGTATCCACCCTATGGGTTGGC-3′

Restriction digestion is carried out at 37 °C for 6 h, samples run on an ethidium bromide stained 2% agarose gel. PCR amplification product is a 603 bp fragment and restriction with Bgl II gives rise to two fragments of 203 and 400 bp. The presence of three bands shows heterozygosity, the 603 band alone shows homozygosity for the C nucleotide and the two bands of 203 and 400 show homozygosity for the T nucleotide. Direct nucleotide sequence analysis of the TCR polymorphism is performed following PCR amplification on both strands (ABI PRISM 310 sequencer) using Big Dye kit as fully described elsewhere [25].

### 2.4. Statistical Analysis

Sex-related differences between genotype distributions were assessed by means of meta-analysis. The meta-analysis compared all the males against all females. Review Manager software (RevMan Version 5.3.5 Copenhagen: The Nordic Cochrane Centre, The Cochrane Collaboration, 2014) was used to examine heterogeneity, estimate the effect size, and detect publication bias. The results of the combined trials were tested using both the Chi-square test and heterogeneity I^2^ test. Substantial heterogeneity was defined as *p*-value < 0.05, I^2^ > 50%. Effect size estimates, expressed as Odds Ratios and 95% Confidence Intervals (CI) were analyzed using fixed-effects models if the data were homogeneous, otherwise random-effects models were used. Chi-square analysis was also performed to investigate differences in the distribution of the three genotypes between males and females. Statistical significance was set at *p* ≤ 0.05.

## 3. Results

The six studies included for re-analysis are described in Table 1 [16,17,18,19,23]. They reported data from 1312 individuals (456 were males and 856 females), all Caucasian of European origin, having an age range between 18 and 51 years old. Of these individuals, 1172 were healthy, while 70 were patients affected by primary biliary cirrhosis and 70 (females) by endometriosis. One study, dealing with endometriosis [26], had just the female group, thus it was not included for the re-analysis.

Heterogeneity among studies was very low (I^2^ = 0.0, *p* = 0.85), so the studies could be considered homogeneous. The genotype distribution of the individuals included in the meta-analysis is shown in Table 2. A total of 49% were common genotype T/C heterozygotes, 51% were homozygotes divided by homozygous status for the two digestion fragments (T/T, 25.5%), or for the 603-bp fragments (C/C, 25.5%).

**Figure 1:**
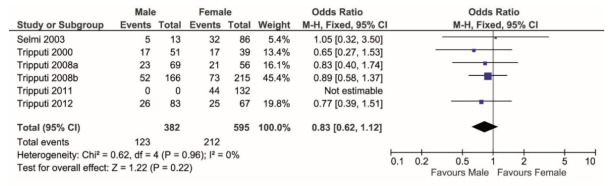
Forest plot comparing the percentage of T/T genotype (events) over the T/T + T/C (total) in males versus females. T/T shows higher frequency in females.

The genotype frequency was significantly different between sexes (Chi-squared = 5.74, correlation = 0.16, df = 2, *p* = 0.05). In men, 57% was heterozygous while 43% was homozygous; in women, 45% was heterozygous while 55% was homozygous (Chi-squared = 2.88 correlation = 0.11, df = 1, *p* = 0.09). Thus, comparing only the two genotype groups, i.e., heterozygous (T/C) versus homozygous (T/T + C/C), females showed higher frequency of homozygosis than males (Chi-squared = 17.3, df = 1, *p* < 0.0001). Considering the homozygosity T/T as compared to T/T + T/C, the former was more prevalent (but not significantly) in males than in females (*p* = 0.22, Figure 1).

**Figure 2:**
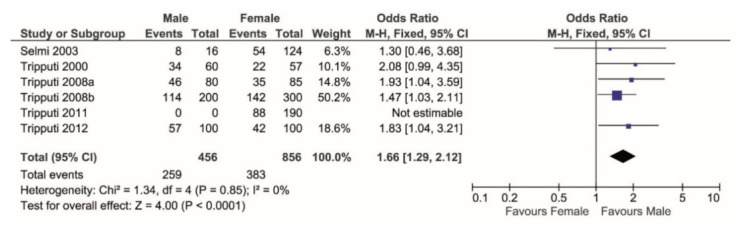
Forest plot comparing the percentage of T/C genotype (events) over the TC + TT + CC (total) in males versus females. T/C shows higher frequency in females.

When considering heterozygosity over total cases, the meta-analysis showed that the T/C genotype is significantly more prevalent in males (*p* < 0.0001, Figure 2).

**Figure 3:**
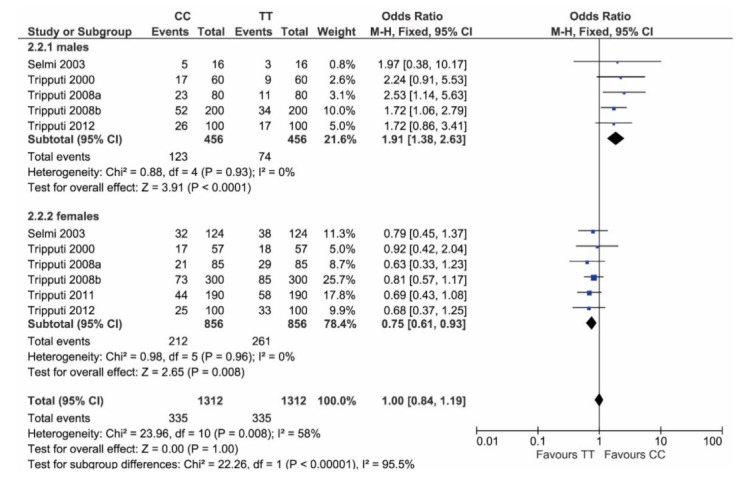
Forest plot comparing homozygotes genotypes (T/T and C/C) in males versus females. In males (plot above), C/C has higher frequency than T/T, while, in females, T/T has higher frequency than C/C.

Post hoc power analysis for dichotomous data and independent samples, considering alpha = 0.05, provided a power value of 98.7%. Considering just homozygosity (T/T versus C/C), the genotype distribution in females again differed from males: males show a significantly higher frequency of C/C (*p* < 0.0001), while females show a significantly higher frequency of T/T (*p* = 0.008) (Figure 3).

## 4. Discussion

This work aims at describing the distribution of the allelic frequencies of a specific SNP in the promoter region of the T cell receptor β gene (rs1800907) from a sex-related perspective. The role of T cells in immune reactivity is greatly associated with the T cell receptor (TCR) loci, which are in turn crucial candidates for assessing common disease susceptibility within the immune system, such as asthma, atopy and autoimmune disorders. Sex-specific genetic architecture has been proposed in the last decades as being pivotal in regulating immune systems. Cell environment in males and females, under the effects of different hormonal levels, correlates with very different gene-environment interactions, greatly impacting on gene mapping of complex traits [1,27,28,29,30]. Recent applications of high-throughput sequencing techniques have allowed the ascertainment of TCR repertoire diversity in humans [8,31,32].

Our re-analysis of the distribution of the genotypes related to the SNP rs1800907 shows that in females, but not in males, there is a significant increase of C/C homozygous status and a proportionate decrease of T/C heterozygous status. Selective pressures [33] and different recombination rates between males and females seem to be the possible mechanism of these findings, although this needs to be further elucidated. Hypothetically, the significant increase of C/C homozygous genotype, as well as the significant decrease of the T/C heterozygous genotype, in females might be responsible of the higher predisposition of women for some T cell-mediated autoimmune disorders. Potentially autoreactive T cells are negatively selected in infant thymus, but in the presence of certain TCR polymorphisms, a certain degree of foreign auto-antigen cross-reactivity may occur; autoreactive T cells with these TCR variants may be less negatively selected [34]. However, the relationship between TCR variants and autoimmune disorders is highly controversial. Some authors have suggested that rs1800907 SNP was associated with autoimmune disease as insulin-dependent diabetes, autoimmune hepatitis [12], IgA nephropathy [13], membranous nephropathy [14], Graves’ disease and Hashimoto’s thyroiditis [15]. However, further reports, associating TCRβ polymorphism and autoimmune diseases, failed to support the link described in the 1990s. They revealed a lack of reproducibility likely due to several reasons, i.e., population stratification, publication bias, effect heterogeneity, lack of statistical power [22] and, as suggested by Payami et al. [35], the need for a better selection of study participants, paying particular attention to age. Nonetheless, the different genotype frequency between males and females among Caucasoid control populations, demonstrated in large samples, could be one of the mechanisms explaining the lack of consistency among reports associating TCRβ polymorphism and autoimmune diseases. In primary biliary cirrhosis, always considering the rs1800907 SNP of the TCRβ promoter region, a less severe disease occurred in heterozygous patients (T/C), as shown by Mayo score, a validated prognostic index (5.1 ± 1.2 vs. 5.7 ± 1.2 in not heterozygous) [19]. T/C heterozygosis was also more prevalent in early disease when compared to C/C homozygosis (11 vs. 31% respectively; *p* = 0.032); in particular, heterozygous patients were significantly younger and had less advanced disease, as highlighted by earlier histologic stage and lower values of the Mayo score [19]. In addition, we found that the polymorphism rs1800907 was not related with endometriosis in an Italian population [26].

The findings of this retrospective re-analysis should be considered with caution, considering that all the included studies were all from the same group of authors, thus reducing the external validity. We directly analyzed the independent cohort coming from the 1000 genomes data project for confirmation (publicly available at: https://www.internationalgenome.org/category/data-access/ accessed on 25 February 2021). The comparison was performed between the dataset of the present study and the dataset of the European population derived from the 1000 genomes database (excluding the Finnish population), which included 404 healthy subjects (202 males and 202 females). The Pearson Chi square test was applied. We found a statistically significant difference in the allelic distribution both within the male group (*p* = 0.006) and within the female group (*p* = 0.02), while, considering the overall population (males and females together), no differences could be found (*p* = 0.78). However, comparing males and females with each other, in the European population, no difference between males and females could be detected in terms of allelic frequency (*p* = 0.40). Notably, while the European population was perfectly balanced in the F: M ratio, in our data the F: M ratio was 1.53. The pooled sample having females more represented than males (856 versus 456), does indeed represent a study pitfall, which correlates with the female bias, characterizing gender differences in autoimmunity.

Further study limitations include the low number of studies included (*n* = 6), published by same authors and concerning just a specific geographical provenance.

In a clinical perspective, this study enhances the need for further investigations to explain the higher women predisposition for some T cell-mediated autoimmune disorders, and supports the need for a sex-matched control group in designing association studies. The significant increase of C/C homozygous genotype as well as the significant decrease of T/C heterozygous genotype in females might be responsible of the higher women predisposition for some T cell-mediated autoimmune disorders.

## 5. Conclusions

Within the limitations of this meta-analysis of data, allelic distribution according to sex might account the role of TCRβ-related SNPs in autoimmune diseases. Further investigations will explain better this genetic background, in the perspective of a sex-related T cell immune responsiveness and auto-immunity.

## Figures and Tables

**Table 1:** Description of enrolled individuals from the six included studies (SD = standard deviation).

Reference	Males	Females	Total Enrolled Individuals
Tripputi and Bignotto 2012 [23]	100	100	200—only healthy controls
Tripputi et al., 2011 [26]	0	190	190—70 cases and 120 controls
Tripputi et al., 2008 [17]	80	85	165—only healthy controls
Tripputi et al., 2008 [18]	200	300	500—only healthy controls
Selmi et al., 2003 [19]	16	124	140—70 cases (62 females and 8 males) and 70 healthy controls (62 females and 8 males)
Tripputi et al., 2000 [16]	60	57	117 (only healthy controls)
Total	456	856	1312 (140 cases and 1172 healthy controls)

**Table 2:** Difference of genotype frequency between males (*n* = 456) and females (*n* = 856) of rs1800907 SNP of the human TCRβ. The total number of individuals included was 1312.

Individuals	Males (Number and %)	Females (Number and %)	Overall (Number and %)
**T/C ^a^**	259 (57%)	383 (45%)	642 (49%)
**T/T ^b^**	123 (27%)	212 (25%)	335 (25.5%)
**C/C ^c^**	74 (16%)	261 (30%)	335 (25.5%)

^a^ Heterozygous; ^b^ Homozygous (two digestion fragments); ^c^ Homozygous (603-bp fragments).

## Data Availability

No new data were created in this study since this is a retrospective meta-analysis of previous studies.
